# Unfavorable Individuals in Social Gaming Networks

**DOI:** 10.1038/srep17481

**Published:** 2015-12-09

**Authors:** Yichao Zhang, Guanrong Chen, Jihong Guan, Zhongzhi Zhang, Shuigeng Zhou

**Affiliations:** 1 Department of Computer Science and Technology, Tongji University, 4800 Cao’an Road, Shanghai 201804, China; 2 Department of Electronic Engineering, City University of Hong Kong, 83 Tat Chee Avenue, Kowloon Hong Kong SAR, China; 3 Department of Computer Science and Engineering, Fudan University, Shanghai 200433, China; 4 Shanghai Key Lab of Intelligent Information Processing, Fudan University, Shanghai 200433, China

## Abstract

In social gaming networks, the current research focus has been on the origin of widespread reciprocal behaviors when individuals play non-cooperative games. In this paper, we investigate the topological properties of unfavorable individuals in evolutionary games. The unfavorable individuals are defined as the individuals gaining the lowest average payoff in a round of game. Since the average payoff is normally considered as a measure of fitness, the unfavorable individuals are very likely to be eliminated or change their strategy updating rules from a Darwinian perspective. Considering that humans can hardly adopt a unified strategy to play with their neighbors, we propose a divide-and-conquer game model, where individuals can interact with their neighbors in the network with appropriate strategies. We test and compare a series of highly rational strategy updating rules. In the tested scenarios, our analytical and simulation results surprisingly reveal that the less-connected individuals in degree-heterogeneous networks are more likely to become the unfavorable individuals. Our finding suggests that the connectivity of individuals as a social capital fundamentally changes the gaming environment. Our model, therefore, provides a theoretical framework for further understanding the social gaming networks.

In recent studies of game theory, two focal topics attract most attention. One is to find the origin of cooperation in the structured populations, such as social gaming networks^1^,^2^,^3^,^4^,^5^,^6^,^7^,^8^,^9^,^10^,^11^,^12^,^13^,^14^,^15^,^16^,^17^,^18^,^19^. The other is to determine whether a strategy updating rule will succeed in a unstructured population^20^,^21^,^22^,^23^,^24^. As of now, the studies on which strategy updating rule is better in social networks^25^,^26^,^27^,^28^ are rather limited. In a well-mixed population^29^ or an unstructured population^22^, a better strategy in a two-player iterated game, such as the recently proposed ‘zero-determinant’ strategy^20^,^21^,^22^, can neither guarantee its dominance in the population nor existence in the population as an ‘evolutionarily stable strategy’ (ESS)^22^,^24^. In social networks^25^,^26^,^27^,^28^, however, a strategy updating rule alone can hardly determine whether it is dominant or an ESS. A dominant strategy updating rule in the unstructured population may be dominated in social networks, if the individuals adopting this strategy do not possess enough social capital^30^. In the social networks, the social capital can be represented by an individual’s topological property.

In this paper, we investigate the unfavorable individuals (UI) in social gaming networks. The UIs are defined as the individuals gaining the lowest average payoff in a round of game. Since the average payoff is usually defined as an individual’s fitness^22^, the UIs are very likely to be eliminated or change their strategy updating rules in evolutionary games guided by Darwinian selection^31^. Thus, further understanding the topological property of the UIs is nontrivial. In this paper, the UIs are not defined by the accumulated payoff, since otherwise the less-connected individuals’ fitness is simply proportional to their degrees from a mean-field prospective. In this scenario, the evaluation system based on the accumulated payoff is apparently unfair to the less-connected individuals. On the other hand, the UIs are different from the ‘dead’ individuals in the Moran process^7^,^32^. In the Moran process, a randomly picked neighbor of a selected individual with a high payoff will be eliminated in each round^7^. Thus, the ‘dead’ individual is eliminated by a high-fitness neighbor, while the UIs are eliminated for their own low fitness.

To keep consistent with previous works^3^,^4^,^5^,^6^,^7^,^8^,^9^,^10^,^11^, we investigate the UIs in a standard non-cooperative game called the Prisoner’s Dilemma (PD). In a 2 × 2 game, *i*'s strategy against *j* is denoted by 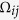, which takes vectors 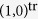 and 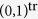 for the cooperative and defective strategies, respectively. For convenience, 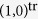 and 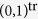 are denoted by 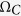 and 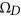 hereafter. In one round of the game playing with the other individual *j*, the payoff 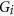 can be rewritten as


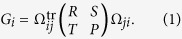


For the PD game, *R*, *S*, *T* and *P* in Eq. 1 are normally set to satisfy two inequalities: 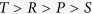 guarantees that the Nash equilibrium of the game is mutual defection, whereas 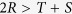 makes mutual cooperation the globally best outcome^20^,^21^,^22^.

For structured populations, on the other hand, most existing game models^3^,^4^,^5^,^6^,^7^,^8^,^9^,^10^,^11^ are based on an assumption that all individuals adopt a unified strategy to play with their neighbors. Recently, inspired by the interesting behavior in public good games^33^, Wardil *et al*. proposed a game model in which each individual adopts simultaneously different strategies against different opponents^34^. In the model, the strategy updating rule is based on the respective accumulated payoffs of the two players on a link. Respecting the strategy updating rule, a recent result in evolutionary game theory suggests that rational individuals only need their experience in the last round to update their strategy to a neighbor^20^,^21^. When playing with a neighbor, an individual’s experience in the last round must be one of the four possible cases, namely, cooperating with a cooperator (CC), cooperating with a defector (CD), defecting a cooperator (DC), and defecting a defector (DD).

Inspired by the probing works mentioned above, we propose a divide-and-conquer game model where individuals play independent games with their neighbors. They have two strategies, to cooperate or to defect. To each neighbor, an individual adopts a particular strategy. For all neighbors, therefore, the individual has a pure strategy set, which is randomly initiated with a certain cooperative probability. We call this probability “the original cooperative will”. In this scenario, we set all the individuals to adopt the same strategy updating rule based on the experience of the last round to guarantee that the gaming environments are fair to them. Thus, the only difference among individuals is their topological property. We will show that this small difference fundamentally changes their fortune.

## Results

### Unfavorable individual distribution in social gaming networks

In a degree-heterogeneous network, such as ‘Facebook’, ‘Twitter’, ‘Flickr’, scientific collaboration networks^35^,^36^,^37^,^38^, among others, degree distribution 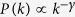, where *γ* is normally confined to interval^2^,^3^ (see^25^,^26^). Given such a degree distribution, less-connected individuals are significantly more than highly-connected individuals. From a mean-field perspective, the average expected payoff should be identical for all the individuals. Considering that neighbors’ strategies of each individual can’t be evenly initiated, the failure of an individual should be the outcome of stochastic fluctuations. Thus, the degree distribution of the UIs should be the same as the degree distribution of the network. Actually, they are rather different. In social gaming networks, the probability of a UI having degree *k* is given by


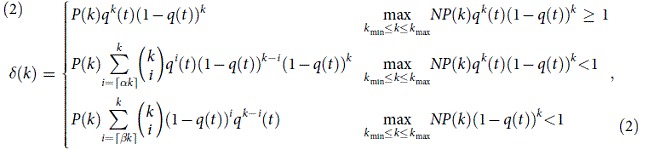


where *α* and *β* (*α*, 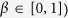 are two finite-size factors, which are less than 1 in small networks. For scale-free networks^25^,^26^, *k*_min_ is the only solution to


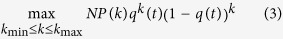


and


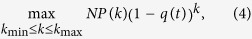


which is the minimum degree of the network. For large networks, where 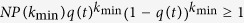, the UI may receive the lowest average payoff *S* (see Eq. 1 in Methods). In smaller networks, conversely, 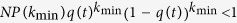. In this case, a UI with the lowest average payoff *S* may not exist, since 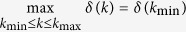 is too small. One then needs to adjust *α* to raise 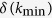 as shown in the second row of Eq. (2). For a certain *N*, *α* can be derived from


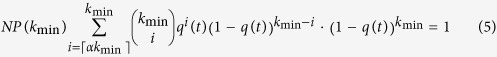


to make Eq. (2) applicable to the relatively better cases, in which the UI’s average payoff may be higher than *S*. If the size of the network is so small such that 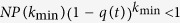, a proper *β* is needed, which can be obtained from


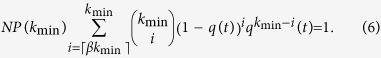


In Eq. (2), the factor 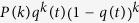 for a less-connected individual is clearly higher than that of his/her highly-connected counterparts. This feature brings a sort of latent but significant difficulty to the less-connected individuals, which has not been reported in the literatures yet. Given Eq. (2), one can derive the degree distribution of UIs from


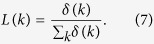


In a degree-homogeneous network, such as the the Watts-Strogatz small-world network (WSSN)^39^, one can also derive *L*(*k*) from Eq. (2). Note that the degree distribution of the WSSN is


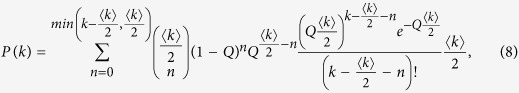


where *Q* denotes the rewiring probability of links^40^. For large networks, where 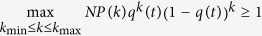, one has 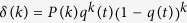. Instead, for 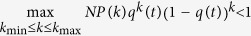, one needs to first find a solution to 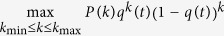. Let 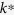 be the solution, which is clearly determined by 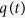 and 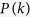. With certain 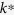 and *N*, *α* can be derived from


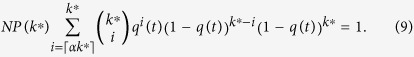


In a similar way, for 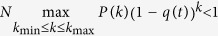, one needs to first find the solution 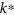 to 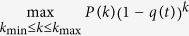. With 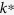 and *N*, *β* can be derived from


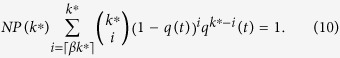


With *β*, one can derive 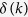 and 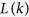 at last.

For the PD game, an individual receives the lowest average payoff when s/he cooperate and all his/her neighbors defect in one round. For the snow-drift game (also known as the hawk-dove or chicken game)^41^,^42^,^43^,^44^, an individual receives the lowest average payoff when s/he suffer the mutual defection in every game with his/her neighbors. Thus, the probability of a UI having degree *k* is given by 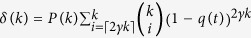. In this scenario, the way of identifying *γ* is simpler than that of identifying *α* and *β* in the PD game, while the expression is not so intuitive as that in the PD game. In the above equation, the term 2*γk* can effectively adjust the UI’s condition from the worst case, *γ* = 1, to better cases, 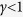. Let 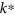 be a solution to 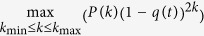, one can derive *γ* from 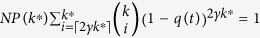. In the Barabási-Albert scale-free network (BASN)^45^, the less-connected individuals are likewise trapped in an unfavorable condition. In a WSSN, however, the least-connected individuals are protected in a statistical sense, since their portion in the population is considerably small.

### Numerical experiments

To clarify the behavior mentioned above and verify our analytical results, we run extensive simulations on the BASNs and WSSNs. We initially generate a network of 1,024 individuals. In the network, we randomly set 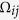 to 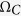 and 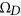 with a certain probability 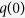 and 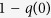, respectively. Note that 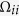 (*i* = 1, 2, …, *N*) will never be used, since self-connection doesn’t exist in these networks. Thus, we uniformly set 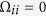. With Eq. (11), we can derive 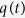 at a certain time step. Inserting 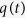 into Eq. (2), we can derive 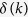. Finally, the degree distribution of UIs is given by Eq. 7. To be consistent with the previous studies, we adopt the PD game as the game model. The parameters of the game, *T*, *R*, *P*, *S*, are set to 1.5, 1, 0, and −0.5, respectively.

Recently, Press and Dyson proposed an interesting strategy updating rule, called the zero-determinant (ZD) strategy^20^,^21^,^22^. This strategy updating rule enables each individual to unilaterally extort its opponent’s payoff in an iterated 2 × 2 game. The extortion is implemented by building an enforced connection between two individuals’ payoffs. This strategy updating rule is a current focal topic in the communities of game-theory researchers^21^, although probabilistic memory-one iterated game is not a new topic^46^,^47^,^48^.

For the rational population with ZD strategies, we test two scenarios: 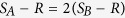 and 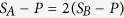, respectively. 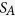 and 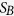 denote A and B’s payoff in each game, respectively. 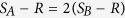 guarantees that individual A can obtain a higher payoff than B in mutual cooperations. 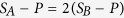 guarantees that individual A can obtain a higher payoff than B in mutual defections. Since the values of 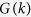 are identical for all *k* in the stationary states of the two scenarios, we measure 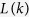 from the beginning of the evolution so as to investigate the transitional states. Details about the ZD strategies and the corresponding simulation settings are shown in the Supplementary Information.

Figure 1 shows the results of both scenarios in a BASN. In the BASN, 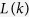 decays quickly with *k* monotonously. The individuals with small degrees, in particular the least-connected ones, are more likely to be UIs. This behavior is striking, since the expected average payoff for all the individuals is the same from a mean-field perspective, namely, 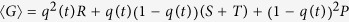. Why the less-connected individuals are more likely to be UIs? This unexpected behavior can be well explained by the analytical solutions of Eq. (2). For a certain 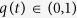, 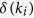 is much greater than 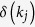 if 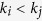. The behavior is fading as 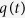 approaches 0 or 1. Note that the disadvantage of less-connected individuals universally exists in various social gaming networks, which doesn’t depend on the strategy updating rule. Thus, the behavior shown in this paper is just a paradigm.

**Figure 2:**
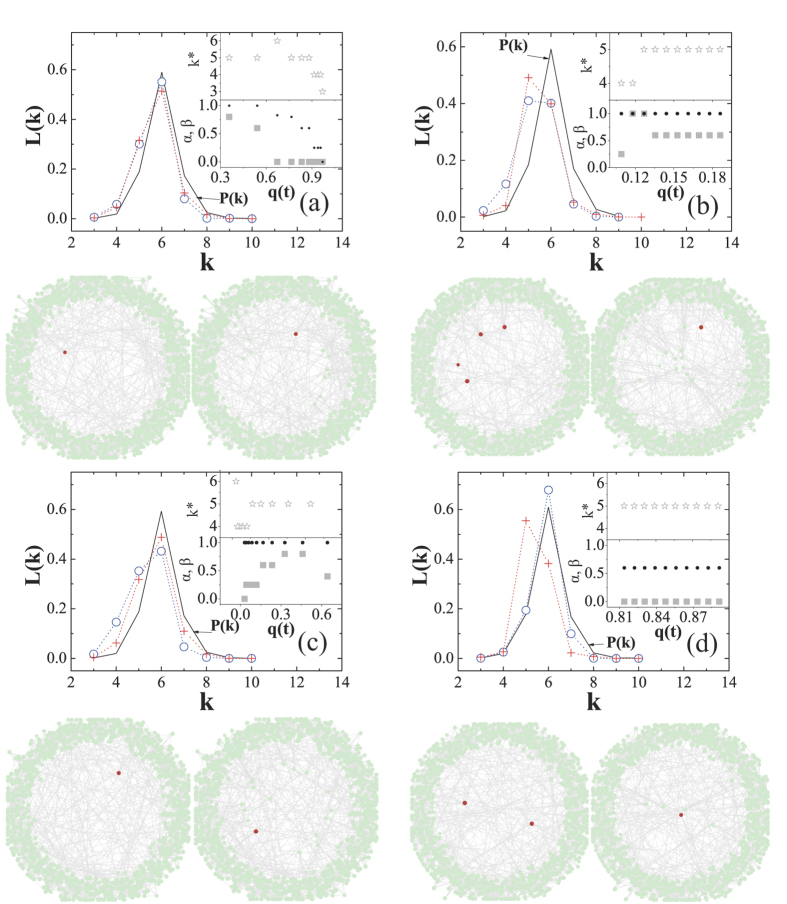
UI distributions in the WSSN. (**a**,**b**) show the simulation results (circles) of 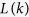 for 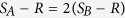 with 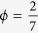 and 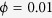, respectively. (**c**,**d**) show the simulation results (circles) of 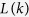 for 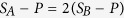 with 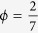 and 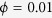, respectively. The crosses correspond to the analytical solutions of Eq. (2). In the insets, solid squares and circles denote *α* and *β*, respectively. Hollow stars denote 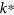. The WSSNs are generated by randomly rewiring 10% (*Q* = 0.1) of the links in the initially regular networks, which are formed by 1,024 identical individuals of degree 6. Note that the average degree of the WSSN equals that of the BASN with *m* = 3. In the WSSN, the individual degrees are distributed in the range 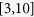. The simulation settings of games are consistent with Fig. 1.

In a WSSN, the number of the less-connected UIs, in 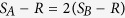 with 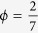, is slightly more than their highly-connected counterparts, as shown in Fig. 2. Interestingly, more less-connected individuals become UIs in (b). This behavior originates from the fact that the values of 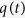 are closer to 0 in (b). Since the gap between 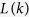 and 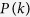 decays with 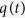, it should be rather small when 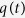 approaches 0. Admittedly, the mean-field approximation provides an analytical solution to 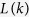, while it also brings some observable deviations as shown in (d). Although protected by the degree-homogeneity, the less-connected individuals in the WSSN are still in greater danger if the system has to eliminate the UIs.

**Figure 3:**
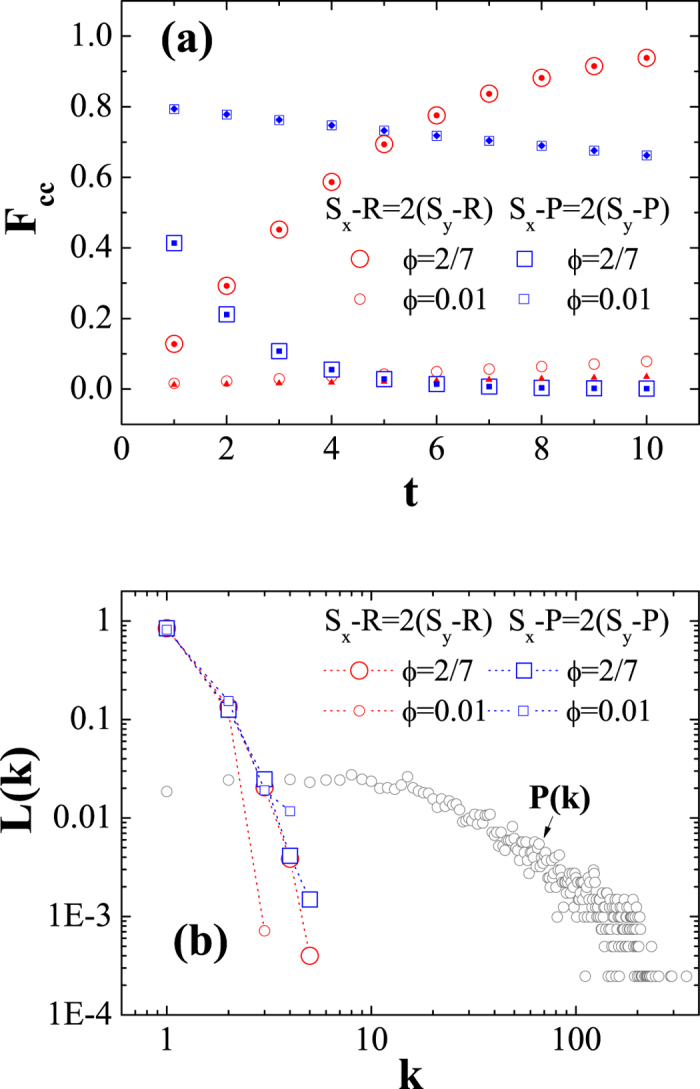
Average frequency of reciprocal pairs and UI distributions in the ‘Facebook’ network. (**a**) shows the evolution processes of the average frequency of reciprocal pairs *F*_*cc*_ for both cases in the ‘Facebook’ network. (**b**) shows the simulation results of 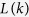 for 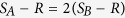 and 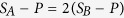 in the ‘Facebook’ network. Solid circles and triangles (squares and diamonds) are the square of the analytical solutions of Eq. (12) (Eq. (13)) in Methods with 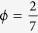 and 0.01, respectively.

**Figure 4:**
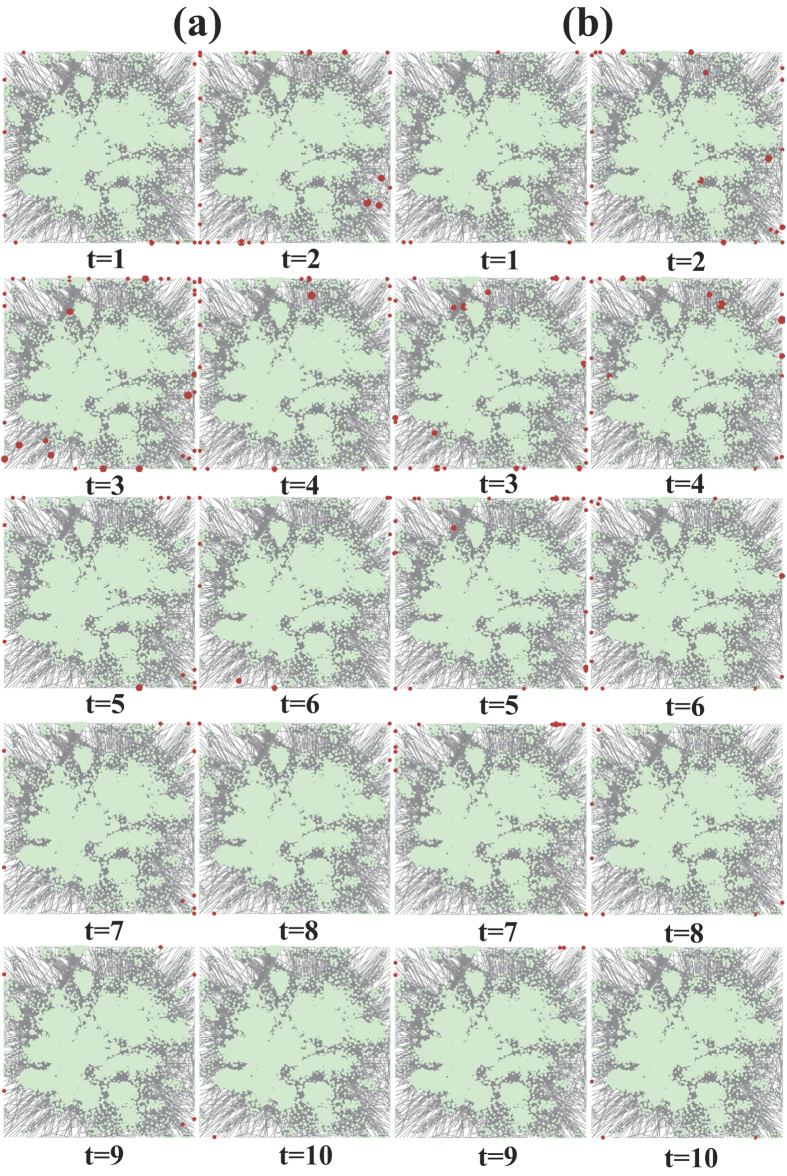
Visualizations of UIs in the iterated PD with the ZD strategies in the ‘Facebook’ network. The UIs are denoted by red circles. (**a**,**b**) show the UIs in the ‘Facebook’ network for 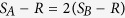 and 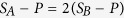 with 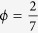, respectively. The size of a circle is proportional to an individual’s degree. Note that the sizes of the UIs are magnified 5 times since a large number of circles are overlapped. The simulation settings of the games are consistent with Fig. 3.

To test our conclusion in real social networks, we run extensive numerical simulations on a real social network based on a data set of ‘Facebook’^49^. Figure 3 shows the results of both scenarios in the Facebook network. 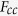 denotes the average frequency of reciprocal pairs. Figure 3(a) shows the evolution of 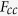. The two ZD strategies lead to two completely different outcomes. 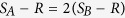 leads the population to a stationary state full of mutual cooperations, while 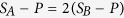 leads it to mutual defections. For both scenarios, 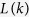 decays quickly with *K*. Figure 3(b) shows the UI’s distribution in the ‘Facebook’ network. One can observe that the less-connected individuals are likewise more likely to be the UIs, although the head of the degree distribution 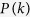 is more flattened than that in the BASN. Figure 4 visualizes the UIs in one round of Monte-Carlo simulation on the ‘Facebook’ network for both scenarios with 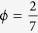, respectively. In Fig. 4, the size of a circle represents an individual’s degree. One can observe that the UIs’ sizes are typically small. Thus, one can hardly observe them without zooming in. This observation intuitively confirms the statistical results shown in Fig. 1.

## Disscusions

Since the evolutionary game theory becomes a focus in the studies of social networks, much effort has been devoted to finding the origin of the widespread cooperative behaviors. For an individual, however, mindless cooperating with his/her neighbors in a social network can hardly guarantee a high payoff in fierce competitions. Thus, one has to understand how to survive in the network first. In terms of social network structures, dynamical social gaming networks are closer to real scenarios^50^ and beneficial to cooperative behaviors^51^. Nevertheless, the system in question evolves too fast compared to the evolution of social networks, then there is no need to model it as a dynamical or temporal network^52^.

After investigating the disadvantage of the less-connected individuals in the social networks with degree heterogeneity, one may see that they are also more likely to receive the highest average payoff. The polarization of their average payoffs indicate that the lonely souls face not only challenges but also chances in social gaming networks. Thus, they may be invaded by the strategy updating rule of hubs and simultaneously invade the hubs if their fitness are based on their average payoffs. This interesting behavior happens to explain why the hubs’ strategy updating rules can hardly keep invariant although they are not UIs.

In a nutshell, we have proposed a divide-and-conquer game model. In this model, each individual possesses a specific strategy to interact with a neighbor in the network. After each round of the game, individuals update their strategies following a certain rule. In this scenario, our analytical and simulation results surprisingly reveal that the less-connected individuals in a degree-heterogeneous network are more likely to become the unfavorable individuals, who are the individuals gaining the lowest average payoff in a round of game.

In the scenarios investigated in this paper, the strategy updating rules, initial conditions, and evaluation criteria are designed to guarantee that the gaming environments are fair to all the individuals. From a mean-field perspective, the expected average payoff for each game between two connected individuals should be identical. Our results, however, reveal that less-connected individuals are always trapped in an unfavorable condition. Since most existing social networks possess a strong degree heterogeneity, an individual’s topological properties in such a network may determine whether s/he can survive in fierce competitions. This disadvantage may be further amplified by his/her smaller degree if the evaluation criteria is based on an individual’s accumulated payoff, which is the sum of payoffs gained in each round of the game. Thus, the drastic growth of individual connectivity in social networks seems to be a consequence of Darwinian selection.

As a social capital, the impact of the individual connectivity on reciprocal behaviors has been extensively discussed in the existing literatures. Its influence on individuals’ fitness has not been analytically investigated as far as we concern. Our analytical results indicate that the difference of the connectivity brings the individuals in the social gaming networks with high degree heterogeneity a remarkable but invisible inequity. Thus, we believe that our model should provide a more realistic theoretical framework for understanding the influence of complex topologies on social cooperation and competition. Our analytical techniques may also be helpful for further analytical studies on social gaming networks. In addition, our conclusion provides a new perspective to the understanding of the evolution of social networks in general.

## Methods

### The divide-and-conquer game model

We take the iterated Prisoner’s Dilemma (IPD) game as example. In the IPD, individuals have two strategies: to cooperate or to defect. To each neighbor, an individual adopts a particular strategy. For all neighbors, therefore, the individual has a strategy set, which is randomly initiated with a certain cooperative probability. We call this probability ‘the original cooperative will value’. Each entry of an individual strategy set evolves respectively with time, following a certain strategy updating rule. Assume that an individual has *k* neighbors and plays a game with each neighbor. In each round of the game, the individual has to play a total of *k* times of the game with its *k* neighbors. We define 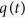 as the average frequency of cooperation in all the strategy sets. Clearly, 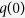 equals the original cooperative will value. If a social network is degree-heterogeneous^25^, one can take it as a heterogeneous organization of two-player pairs. For each two-player pair, two individuals simultaneously update their strategies based on their experience gained from the previous round of the game^20^,^21^,^22^,^53^,^54^,^55^,^56^.

When two connected individuals play a game, each individual has to experience one of the four possible cases, namely, cooperating with a cooperator (CC), cooperating with a defector (CD), defecting a cooperator (DC), and defecting a defector (DD). We define a strategy updating rule 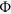 by 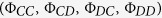, which is a vector composed of the probabilities of cooperation after experiencing each of the four cases, respectively. For different individuals, their updating rules should be different, while we set them to the same to guarantee that they are equally intelligent. With a mean-field approximation, the evolution of 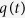 satisfies the following equation:


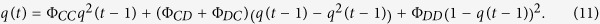


If individuals uniformly adopt the celebrated ‘tit-for-tat’ rule^53^, then 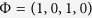. Since they have to cooperate at the beginning, 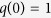. If their first moves are randomly assigned by the original cooperative will value, these tit-for-tat individuals will keep backbiting. The strategy matrix of the individuals will be transposed back and forth. Thus, 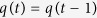 in this case. If the individuals uniformly adopt the ‘Pavlov’ rule (also known as the ‘win-stay-lose-shift’ rule^57^), 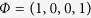. In this case, 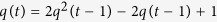, which grows drastically with time and quickly reaches the maximum 1. Two simple examples are shown in Table 1.

In the ZD strategy, 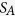 and 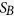 denote an individual and his/her opponent’s expected payoff, respectively, in an iterated 2 × 2 game. When 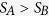, A’s payoff is expected to exceed B’s payoff. For 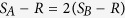, one has


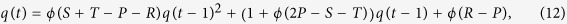


where 
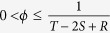
 (details are shown in the Supplemental Material). In this case, 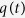 grows monotonously with *t*. The rate of growth depends on 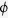, and 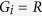 for all *i* in the stationary state. For 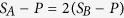, one has


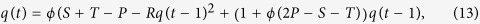


where 
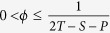
. In this case, 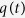 decays monotonically with *t* and 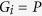 for all *i* in the stationary state. Since the ZD strategies lead the evolution of cooperation to two opposite directions, we will investigate the UIs in the social gaming networks governed by the two ZD strategies, respectively.

Let the size of a social gaming network be *N*. In the strategy matrix Ω, an individual *i* has 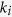 strategies corresponding to his/her 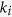 neighbors, where 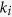 denotes *i*'s degree (namely, number of connections). For convenience, cooperation and defection are denoted by *C* and *D*, respectively. For each pair of connected individuals, Ω_*ij*_ takes Ω_*C*_ or Ω_*D*_ with a probability 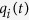 or 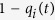, respectively. To create a fair gaming scenario for all the individuals, we set 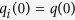 for all *i*, where *i* = 1, 2, …, *N*. Simultaneously, all the individuals adopt the same ZD strategy. Therefore, the only difference among individuals is their topological property in the network. Since social networks are neither regularly nor completely randomly connected, this complex texture covers many unknown inequalities. Through extensive simulations, we observe that the majority of UIs in the BASN^45^ are the less-connected ones, namely, the lonely souls. This special behavior has not been reported elsewhere in the literatures.

## Additional Information

**How to cite this article**: Zhang, Y. *et al*. Unfavorable Individuals in Social Gaming Networks. *Sci. Rep*. **5**, 17481; doi: 10.1038/srep17481 (2015).

## Supplementary Material

Supplementary Information

## Figures and Tables

**Figure 1:**
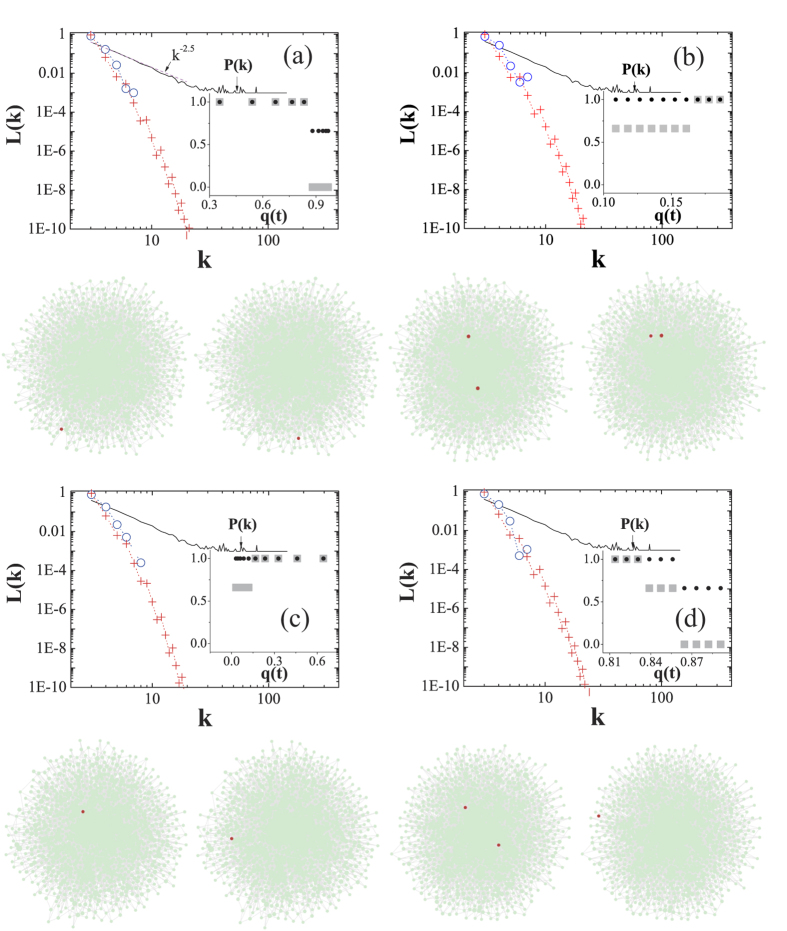
UI distributions in the BASN. (**a**,**b**) show the simulation results (circles) of 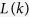 for 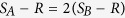 with 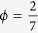 and 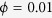, respectively. (**c**,**d**) show the simulation results (circles) of 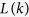 for 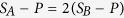 with 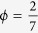 and 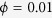, respectively. The crosses correspond to the analytical solutions of Eq. (2). The experiments are visualized in the corresponding panels. The left (right) inset shows the result of the first (last) time step. The UIs are shown in red. In the BASN, the degree distribution should be 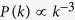 when the size of network is large. Considering the size of the tested network is relatively small, the degree distribution is set to 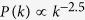 in our mathematical derivations. The dashed line in (**a**) is the fitting curve of the degree distribution 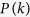 (solid line) of the BASN. One can see that the power exponent of 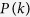 in the tested network is −2.5. In our mathematical deviations, the individuals’ degrees are distributed in the range 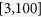. In our simulations, 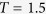, 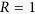, *P* = 0, and 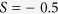. 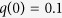 and 0.9 for 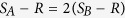 and 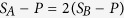, respectively. In this case, the expected average payoff is 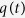. The BASNs are generated by 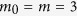
45. Here, *m*_0_ denotes the size of the initial fully-connected network and *m* denotes the number of links among a new node and the existing individuals in the network.

**Table 1:** Evolution of the divide-and-conquer game.

Pavlov: Φ = (1, 0, 0, 1)			
*t* = 1	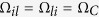	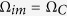 , 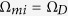	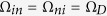
*t* = 2	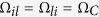	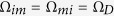	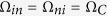
*t* = 3	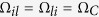	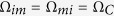	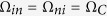
Tit for tat: 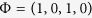			
*t* = 1	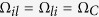	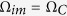 , 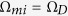	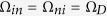
*t* = 2	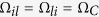	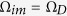 , 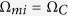	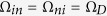
*t* = 3	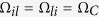	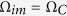 , 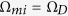	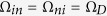

*l*, *m* and *n* are *i*'s neighbors. Since their initial strategies are different, *i* needs to adjust its strategy set accordingly. The evolution of each entry in *i*'s strategy set is independent of the others.
