# Supplementary material for: Unfavorable Individuals in Social Gaming Networks
Source: Sci Rep. 2015 Dec 9;5:17481. doi: 10.1038/srep17481 (PMC4673536; doi:10.1038/srep17481)
Supplement: Supplementary Information [file srep17481-s1.pdf]

Unfavorable Individuals in Social Gaming Networks
Supplementary Information

Yichao Zhang[1],[*] Guanrong Chen[2],[†] Jihong Guan[1],[‡] Zhongzhi Zhang[3],[§] and Shuigeng Zhou[3][¶]
[1]*Department of Computer Science and Technology, Tongji University, 4800 Cao'an Road, Shanghai 201804, China*
[2]*Department of Electronic Engineering, City University of Hong Kong, 83 Tat Chee Avenue, Kowloon Hong Kong SAR, China and*
[3]*Department of Computer Science and Engineering, Fudan University, Shanghai 200433, China Shanghai Key Lab of Intelligent Information Processing, Fudan University, Shanghai 200433, China*

To understand why we choose the ZD strategy [1] as a rational strategy updating rule, we briefly introduce the ZD strategy in the 2×2 iterated prisoner dilemma game here. For each game between two connected individuals, every individual has to experience one of the four possible cases, namely, cooperating with a cooperator (CC), cooperating with a defector (CD), defecting a cooperator (DC), and defecting a defector (DD). We define a state vector Φ by $(\Phi_{CC}, \Phi_{CD}, \Phi_{DC}, \Phi_{DD})$ as the probability of experiencing each of the four cases, respectively. If the strategy updating is a Markov process, we can find a Markov transition matrix M to realize the evolution of the strategy. For two players, A and B, we have

$$M_A = \begin{pmatrix} p_{CC}s_{CC} & p_{CC}(1-s_{CC}) & (1-p_{CC})s_{CC} & (1-p_{CC})(1-s_{CC}) \\ p_{CD}s_{DC} & p_{CD}(1-s_{DC}) & (1-p_{CD})s_{DC} & (1-p_{CD})(1-s_{DC}) \\ p_{DC}s_{CD} & p_{DC}(1-s_{CD}) & (1-p_{DC})s_{CD} & (1-p_{DC})(1-s_{CD}) \\ p_{DD}s_{DD} & p_{DD}(1-s_{DD}) & (1-p_{DD})s_{DD} & (1-p_{DD})(1-s_{DD}) \end{pmatrix}, \tag{1}$$

where the vectors $\mathbf{p} = (p_{CC}, p_{CD}, p_{DC}, p_{DD})$ and $\mathbf{s} = (s_{CC}, s_{CD}, s_{DC}, s_{DD})$ denote A and B's probabilities of cooperation in the next round after experiencing CC, CD, DC, and DD cases, respectively. If i is a completely irrational individual with a constant probability of cooperation q, $\mathbf{p} = (q, q, q, q)$. Since Press and Dyson [1] have demonstrates that a longer memory doesn't bring a player any benefit in an indefinitely iterated 2×2 game, the evolution of a player's state can be considered as a Markovian process. Then the evolution of A's state vector $\Phi_A(t)$ is given by

$$\Phi_A(t) = \Phi_A(t-1)M_A. \tag{2}$$

Considering M_A has a unit eigenvalue, we set a variant matrix $J = M_A - I$, which is singular, namely, $det(J) = 0$. Adding the first column of J into the second and the third columns, respectively, we obtain

$$J' = \begin{pmatrix} -1+p_{CC}s_{CC} & -1+p_{CC} & -1+s_{CC} & (1-p_{CC})(1-s_{CC}) \\ p_{CD}s_{DC} & -1+p_{CD} & s_{DC} & (1-p_{CD})(1-s_{DC}) \\ p_{DC}s_{CD} & p_{DC} & -1+s_{CD} & (1-p_{DC})(1-s_{CD}) \\ p_{DD}s_{DD} & p_{DD} & s_{DD} & (1-p_{DD})(1-s_{DD}) \end{pmatrix}. \tag{3}$$

Since these manipulations consist of elementary column transformations, its determinant equals $det(J)$. Based on Cramer's rule, we have

$$\begin{pmatrix} C_{11} & C_{21} & C_{31} & C_{41} \\ C_{12} & C_{22} & C_{32} & C_{42} \\ C_{13} & C_{23} & C_{33} & C_{43} \\ C_{14} & C_{24} & C_{34} & C_{44} \end{pmatrix} J' = det(J')I = 0, \tag{4}$$

where C_{ij} is the algebraic complement of J'. Given Eq. (4), for the fourth row of the adjugate matrix of J', we have

$$(C_{14}, C_{24}, C_{34}, C_{44})J' = (0,0,0,0). \tag{5}$$

Since $J' = M_A - I$, we obtain

$$(C_{14}, C_{24}, C_{34}, C_{44})M_A = (C_{14}, C_{24}, C_{34}, C_{44}). \tag{6}$$

[*]Electronic address: yiczhang@cs.ucl.ac.uk
[†]Electronic address: gchen@ee.cityu.edu.hk
[‡]Electronic address: jhguan@tongji.edu.cn
[§]Electronic address: zhangzz@fudan.edu.cn
[¶]Electronic address: sgzhou@fudan.edu.cn

Thus, $(C_{14}, C_{24}, C_{34}, C_{44})$ is a stationary vector of M_A. Let $v = (C_{14}, C_{24}, C_{34}, C_{44})$, which is a fixed point in the Markov Chain of $\Phi_A(t)$. For convenience, let A's payoff vector be $W_A = (R, S, T, P)$ and B's payoff vector be $W_B = (R, T, S, P)$. After normalization, we obtain their respective expected payoffs,

$$S_A = \frac{v \cdot W_A}{C_{14} + C_{24} + C_{34} + C_{44}} \tag{7}$$

and

$$S_B = \frac{v \cdot W_B}{C_{14} + C_{24} + C_{34} + C_{44}}. \tag{8}$$

Considering a linear combination of A and B's expected payoffs, we have

$$\begin{aligned}\Delta(p,s) &= \alpha S_A + \beta S_B + \gamma \qquad (9)\\ &= \frac{v \cdot (\alpha W_A + \beta W_B + \gamma(1,1,1,1))}{C_{14} + C_{24} + C_{34} + C_{44}}\\ &= \frac{det\begin{pmatrix} -1 + p_{CC}s_{CC} & -1 + p_{CC} & -1 + s_{CC} & (\alpha+\beta)R + \gamma \\ p_{CD}s_{DC} & -1 + p_{CD} & s_{DC} & \alpha S + \beta T + \gamma \\ p_{DC}s_{CD} & p_{DC} & -1 + s_{CD} & \alpha T + \beta S + \gamma \\ p_{DD}s_{DD} & p_{DD} & s_{DD} & (\alpha+\beta)P + \gamma \end{pmatrix}}{C_{14} + C_{24} + C_{34} + C_{44}}.\end{aligned}$$

Thus, once A's Markovian update rule satisfies

$$\begin{pmatrix} -1 + p_{CC} \\ -1 + p_{CD} \\ p_{DC} \\ p_{DD} \end{pmatrix} = X \begin{pmatrix} (\alpha+\beta)R + \gamma \\ \alpha S + \beta T + \gamma \\ \alpha T + \beta S + \gamma \\ (\alpha+\beta)P + \gamma \end{pmatrix}, \tag{10}$$

a simple linear relation $\alpha S_A + \beta S_B + \gamma = 0$ emerges, where $X \in \Re$ and $X \neq 0$. Hence, this updating rule is called the zero-determinant (ZD) strategy [1, 2].

With a stationary ZD strategy $\mathbf{p}$, we can derive the following difference equation:

$$q(t) = p_{CC}q^2(t-1) + p_{CD}q(t-1)(1-q(t-1)) + p_{DC}q(t-1)(1-q(t-1)) + p_{DD}(1-q(t-1))^2. \tag{11}$$

For $S_A - R = 2(S_B - R)$, $\alpha = \phi$, $\beta = -2\phi$, and $\gamma = R\phi$, where $\phi > 0$. From Eq. (10), we have

$$\begin{pmatrix} p_{CC} \\ p_{CD} \\ p_{DC} \\ p_{DD} \end{pmatrix} = \begin{pmatrix} 1 \\ 1 + \phi(S - 2T + R) \\ \phi(T - 2S + R) \\ \phi(R - P) \end{pmatrix}. \tag{12}$$

Since p_{CC}, p_{CD}, p_{DC}, $p_{DD} \in [0, 1]$, the allowed range of ϕ is $0 < \phi \leq \frac{1}{T-2S+R}$. Let $R = 1$, $S = -0.5$, $T = 1.5$, and $P = 0$, so that $0 < \phi \leq \frac{2}{7}$. Inserting $\mathbf{p}$ to Eq. (11), we obtain

$$q(t) = \phi(S + T - P - R)q^2(t-1) + (1 + \phi(2P - S - T))\, q(t-1) + \phi(R - P). \tag{13}$$

Respecting $R = 1$, $S = -0.5$, $T = 1.5$, and $P = 0$, we obtain the solution $q(t) = 1 + (1-\phi)^t(q(0) - 1)$, which grows monotonically with t. Note that the value of T can be a random value greater than 1, which is irrelevant to the evolution of $q(t)$. Taking the maximum of ϕ, $\frac{2}{7}$, we have $q(t) = 1 + \left(\frac{5}{7}\right)^t (q(0) - 1)$. When $t \gg 1$, $q(t) \simeq 1$. In this case,

$$\mathbf{p} = (p_{CC}, p_{CD}, p_{DC}, p_{DD})^{\text{tr}} = \left(1, \frac{2}{7}, 1, \frac{2}{7}\right)^{\text{tr}}. \tag{14}$$

For completeness, we also test the case that ϕ is close to but still greater than 0. Letting ϕ be 0.01 leads to $q(t) = 1 + (0.99)^t (q(0) - 1)$. In this case,

$$\mathbf{p} = (p_{CC}, p_{CD}, p_{DC}, p_{DD})^{\text{tr}} = (1, 0.975, 0.035, 0.01)^{\text{tr}}. \tag{15}$$

When $S_A - P = 2(S_B - P)$, we have $\alpha = \phi$, $\beta = -2\phi$, and $\gamma = P\phi$, where $\phi > 0$. From Eq. (10), we have

$$\begin{pmatrix} p_{CC} \\ p_{CD} \\ p_{DC} \\ p_{DD} \end{pmatrix} = \begin{pmatrix} 1+\phi(P-R) \\ 1+\phi(S-2T+P) \\ \phi(T-2S+P) \\ 0 \end{pmatrix}. \tag{16}$$

In a similar way, we can derive the allowed range of ϕ, as $0 < \phi \leq \frac{1}{2T-S-P}$. Inserting $\mathbf{p}$ to Eq. (11) yields

$$q(t) = \phi(S+T-P-R)q^2(t-1) + (1+\phi(2P-S-T))\,q(t-1). \tag{17}$$

With $R = 1$, $S = -0.5$, $T = 1.5$, and $P = 0$, we obtain the solution $q(t) = (1-\phi)q(t-1)$. Taking the maximum $\phi = \frac{2}{7}$ gives $q(t) = \left(\frac{5}{7}\right)^t q(0)$. When $t \gg 1$, $q(t) \simeq 0$. Thus, $G_i(\infty) \simeq 0$ in the stationary state. In this case,

$$\mathbf{p} = (p_{CC}, p_{CD}, p_{DC}, p_{DD})^{\mathrm{tr}} = \left(\frac{5}{7}, 0, \frac{5}{7}, 0\right)^{\mathrm{tr}}. \tag{18}$$

Likewise, let ϕ be 0.01, so that $q(t) = (0.99)^t\, q(0)$. In this case,

$$\mathbf{p} = (p_{CC}, p_{CD}, p_{DC}, p_{DD})^{\mathrm{tr}} = (0.99, 0.965, 0.025, 0)^{\mathrm{tr}}. \tag{19}$$

[1] Press W. H. & Dyson F. J. Iterated Prisoner's Dilemma contains strategies that dominate any evolutionary opponent. *Proc. Natl. Acad. Sci. USA* **109**, 10409-10413 (2012).
[2] Stewart A. J. & Plotkin J. B. Extortion and cooperation in the Prisoner's Dilemma. *Proc. Natl. Acad. Sci. USA* **109**, 10134-10135 (2012).
